# Association between device-measured physical activity and lumbar Modic changes

**DOI:** 10.1186/s12891-020-03638-y

**Published:** 2020-09-25

**Authors:** Marella Modarress Julin, Jesperi Saukkonen, Petteri Oura, Maisa Niemelä, Juho-Antti Junno, Juhani Määttä, Jaakko Niinimäki, Timo Jämsä, Raija Korpelainen, Jaro Karppinen

**Affiliations:** 1 Medical Research Center Oulu, Oulu University Hospital and University of Oulu, PO Box 5000, FI-90014 Oulu, Finland; 2 Center for Life Course Health Research, PO Box 5000, FI-90014 Oulu, Finland; 3 Clinic of Physiatry, Oulu University Hospital (OYS), PO Box 21, 90029 Oulu, Finland; 4 Research Unit of Medical Imaging, Physics and Technology, Faculty of Medicine, University of Oulu, PO Box 5000, FI-90014 Oulu, Finland; 5 Cancer and Translational Medicine Research Unit, Faculty of Medicine, University of Oulu, PO Box 5000, FI-90014 Oulu, Finland; 6 Department of Archaeology, Faculty of Humanities, University of Oulu, PO Box 5000, FI-90014 Oulu, Finland; 7 Diagnostic Radiology, Oulu University Hospital (OYS), P.O. Box 10, FI-90029 Oulu, Finland; 8 Department of Sports and Exercise Medicine, Oulu Deaconess Institute Foundation, PO Box 365, FI-90100 Oulu, Finland; 9 Finnish Institute of Occupational Health, Aapistie 1, FI-90220 Oulu, Finland

**Keywords:** Physical activity, MVPA, Device-based measurements, Modic changes, Lumbar spine, Lumbar MRI, Cohort study, Vertebrae, Skeletal health

## Abstract

**Background:**

Modic changes (MC) in the lumbar spine are considered one potential etiological factor behind low back pain (LBP). Multiple risk factors for MC have been suggested, including male gender, smoking and factors affecting hyperloading and mechanical stress such as high body mass index (BMI), strenuous physical work and high occupational and leisure-time physical activity (PA). So far, the effect of PA on the occurrence of MC has remained under debate due to contradictory findings. The purpose of this study was to investigate the possible association between device-measured moderate-to-vigorous PA (MVPA) (≥ 3.5 METs) and lumbar MC.

**Methods:**

The study had 1374 participants from the Northern Finland Birth Cohort 1966. At the age of 46–48, PA was measured by a wrist-worn accelerometer, and lumbar magnetic resonance imaging (MRI) was carried out to determine MC. We analyzed the association between Type 1 (MC1) and Type 2 (MC2) MC and daily amount of MVPA (min/day) using sex-stratified logistic regression models before and after adjustment for BMI, socioeconomic status, smoking, and accelerometer wear time.

**Results:**

Among men, increased amount of MVPA was positively associated with any MC (adjusted OR corresponding to every 60 min/day of MVPA 1.41; 95% confidence interval (CI) 1.01 to 1.95) and MC2 (OR 1.54; 95% CI 1.14 to 2.08), but not with MC1 (OR 1.06; 95% CI 0.80 to 1.39). Among women, we only found a positive association between MVPA and MC1 before adjustments (unadjusted OR 1.42; 95% CI 1.06 to 1.92).

**Conclusion:**

Among men, increased amount of MVPA was associated with increased odds of any MC and particularly MC2. Among women, MVPA was not independently associated with MC.

## Background

It has been suggested that Modic changes (MC) in the lumbar spine are associated with non-specific low back pain (LBP) [1–4], although a recent systematic review showed this association to be inconsistent [5]. MC are lesions of vertebral bone marrow adjacent to vertebral endplates seen in magnetic resonance imaging (MRI) and are further divided into Type 1 (MC1), Type 2 (MC2) and Type 3 (MC3) on the basis of their different appearances in T1- and T2-weighted images [1, 6–8]. It is believed that the same pathologic processes lead to different types of MC. Histological studies show that MC1 is the acute phase, signifying an inflammatory reaction of the bone marrow, whereas MC2 represent fat infiltration and MC3 a sclerotic change of the bone marrow [5–8].

Despite decades of research on MC, their etiology remains unknown [9]. Both mechanical stress and bacterial infection are proposed to play a key role in the development of MC [1, 5, 9]. Age and damage of the endplates and intervertebral discs, such as disc degeneration (DD) and endplate defect, have been consistently associated with MC [9–13]. Multiple other risk factors for MC have also been suggested, including male gender, smoking and factors affecting hyperloading and mechanical stress such as high body mass index (BMI), strenuous physical work and high occupational and leisure-time physical activity (PA) [1, 11, 14, 15].

As regards the association between PA and MC, knowledge is limited and findings are controversial; some results have indicated no association between intense PA and MC [11, 16, 17] whereas others have proposed an association [15]. Somewhat indistinct results have also been reported [14]. The hypothesis behind the association between PA and MC is that a high level of intense PA could cause excessive loading in the motion segment of the spine, i.e. intervertebral disc and adjacent endplates and vertebrae, eventually leading to damage of the disc and endplate followed by chronic inflammation and possibly MC-related edema [9, 18, 19].

As the pathophysiology and etiology of MC and the association between MC and LBP are still under eager debate, the clinical significance of MC also remains controversial [2, 5, 20]. Meanwhile, evidence of a higher prevalence of MC among LBP patients than among the general population [21] warrants a more comprehensive understanding of MC. Thus, investigating the possible risk factors is crucial to obtain a more profound comprehension of the etiology and clinical importance of MC. The objective of this study was to determine whether device-measured moderate-to-vigorous PA (MVPA) is associated with lumbar MC. The hypothesis was that higher amount of MVPA would be associated with lumbar MC.

## Methods

### Study design

The aim of this cross-sectional study was to investigate the possible association between device-measured MVPA and lumbar MC. We used accelerometers to measure PA, and the presence of MC was determined from lumbar MRI scans of the Northern Finland Birth Cohort 1966 (NFBC1966) members, the final study population being 1374 individuals. Presentation of the study setting conform to the guidelines for reporting observational studies (STROBE) [22].

### Selection of study participants from birth cohort population

The collection of Northern Finland Birth Cohort 1966 (NFBC1966) data started in 1965. Pregnant women living in Oulu and Lapland were asked during their maternity clinic appointment to take part in the NFBC1966. The inclusion criterion was that the child’s expected date of birth was between January 1st and December 31st, 1966. Since then, the mothers (*n* = 12,068) and children (*n* = 12,231) have been followed through regular postal questionnaires, clinical examinations and data collected from health care records [23, 24].

The members of the NFBC1966 whose contact information was available and up to date (*n* = 10,321) received postal questionnaires in 2012–2014, and 6825 (66%) individuals aged 46 to 48 at that time responded. In addition, those who lived in Finland were asked to take part in clinical examinations, and 5861 (57%) individuals did so. A trained study nurse measured the height and weight of the participants, and based on these measurement values, BMI (kg/m2) was calculated. The participants were asked about smoking as follows: 1) “Have you ever smoked cigarettes (yes/no)?” and 2) “Do you currently smoke (yes/no)?” Based on the answers, three groups were created: 1) non-smokers, 2) former smokers and 3) current smokers. Socioeconomic status was defined as attended school years (≤ 9 years, 9–12 years, > 12 years) and we specified basic education using response options of: 1) Less than 9 years of elementary school, 2) comprehensive school, or 3) matriculation examination. Furthermore, participants (*n* = 1988) who lived up to 100 km from the city of Oulu received an invitation to undergo lumbar MRI at the Oulu University Hospital. A total of 1540 underwent MRI, but after excluding participants with missing images, imaging modalities, covariates, or PA data, the final sample size was 1374 individuals.

### Physical activity assessment

The PA assessment was part of the NFBC1966 premeditated follow-up organized in 2012–2014. The participants wore a waterproof accelerometer, Polar Active (Polar Electro, Kempele, Finland), which has a 21-day memory, for at least 14 days and 24 h per day, on their non-dominant wrist [25, 26]. Polar Active uses age, sex, height and weight as predefined inputs, and metabolic equivalent (MET) values with an epoch length of 30 s are received [26]. Energy expenditure (EE) during exercise training has been determined, and good correlation (*R*^2^ = 0.74) between the Polar Active and the doubly labeled water technique has been found previously [25, 27]. Participants with at least four valid days were included in the analysis [28]. If at least 600 min/day, i.e. 10 h per day monitoring time during waking hours were recorded, this was included as a valid analysis day. However, the day on which the participants received the accelerometer was excluded. Daily duration averages (min/day) were defined at five activity levels (very light: 1–2 MET, light: 2–3.5 MET, moderate: 3.5–5 MET, vigorous: 5–8 MET and very vigorous: ≥ 8 MET) using the threshold values presented by the manufacturer [26]. We assessed MVPA as all activity at an intensity of at least 3.5 METs (min/day), and converted the values to 60 min/day, i.e. 1 h per day, for the analyses. The MVPA (min/day) range was 5–272 (i.e. 0.08–4.53 h/day) for men and 7–245 (i.e. 0.11–4.09 h/day) for women, whereas wear time (min/day) range was 722–1171 (12.04–19.52 h/day) and 718–1132 (11.97–18.86 h/day) for men and women, respectively. The number of valid days ranged from 4 to 17 for men and 4 to 16 for women.

### Determination of MC from lumbar MRI

Lumbar MR images of the included participants were obtained by the staff of the department of radiology, Oulu University Hospital, using 1.5-T MRI (Signa HDxt, General Electric, Milwaukee, WI) in 2012–2014 (participants aged 46–48). The imaging was performed by T2-weighted fast-recovery fast spin-echo (frFSE) images in the sagittal (repetition time/effective echo time (TR/effTE) 3500/112 ms, 4 averages, field-of-view (FOV) 280 × 280 mm, acquisition matrix 448 × 224, slice thickness 3 mm with 1 mm interslice gap) and transverse planes (TR/effTE 3600/118 ms, 4 averages, FOV 180 × 180 mm, acquisition matrix 256 × 224, slice thickness 4 mm with 1 mm interslice gap) and T1-weighed fluid-attenuated inversion recovery sequence images in the sagittal plane (TR/effTE 860/20 ms, inversion time (TI) of 1969 ms, 1.5 averages, FOV 280 × 280 mm, acquisition matrix 256 × 224, slice thickness 3 mm, interslice gap 4 mm). The scans were accessed using NeaView Radiology software (Neagen Oy, Oulu, Finland), version 2.31.

The evaluation of MC has been previously described elsewhere [4]. The same researcher (JS) analyzed the presence and classification of different types of MC in all the scans using the classification method previously described by Määttä et al. [12]. The intra- and interobserver reliability, determined by Cohen’s Kappa/weighted Kappa, was good for the general presence of MC and the type of MC (for more information, see Saukkonen et al. [4]).

### Ethics

The study protocol followed the Declaration of Helsinki and we obtained the consent of the Ethical Committee of the Northern Ostrobothnia Hospital District. The cohort members participated voluntarily and anonymously; informed consent was signed at each level of the study and recognition of individuals was made impossible by using identification codes instead of personal details.

### Statistical analyses

SPSS version 24 (IBM, Armonk, New York, USA) was used for analyzing the data. The threshold for statistical significance was *P* = 0.05. Descriptive statistics were calculated as means with standard deviations (SD) or medians with interquartile ranges (IQR) for continuous variables, depending on the normality of the data, and as percentages and frequencies for categorical variables. Logistic regression models were used when determining the association between MVPA and lumbar MC. Any MC, MC1 and MC2 were used as separate outcomes, for which we ran crude and adjusted regression models. MVPA (60 min/day, continuous) served as the primary explanatory variable. The following covariates were also included in the adjusted models: body mass index (continuous), socioeconomic status (categorical), smoking (categorical), and accelerometer wear time (continuous). As significant sex interactions were present (*P* values for the sex*MVPA interaction terms in pooled-sex models ≤0.022), all the models were stratified by sex. Odds ratios (OR), their 95% confidence intervals (CI), and the respective *P* values were collected from the data output. Analysis of representativeness was also performed, comparing individuals included in the sample to the rest of the NFBC1966 population. We used the chi-square test for categorial variables, the independent T-test for continuous variables with normal distributions, and the Mann–Whitney U-test for continuous variables with skewed distributions.

## Results

The characteristics of the study population, which comprised 640 men and 734 women, are presented in Table 1. The median amount of MVPA was 73 min/day among men and 57 min/day among women. The prevalence of any MC was 74% among men and 60% among women. As regards the type of MC, 33% of both sexes had MC1, whereas MC2 was observed among 65 and 44% of men and women, respectively. Among men, daily amount of MVPA was positively associated with any MC and MC2, and these associations persisted after full adjustments (Table 2). Among women, we only observed a positive association between MVPA and MC1 before adjustments (Table 3).

**Table 1:** Characteristics of study participants (*n* = 1374) and those excluded from sample (n varies due to missing data)

	Sample		Excluded		P for difference between Sample and Excluded	
	Men (*n* = 640)	Women (*n* = 734)	Men	Women	Men	Women
**Age**, years; mean (SD^a^)	46.8 (0.4)	46.8 (0.4)	46.8 (0.5)	46.8 (0.4)	0.723	0.426
**BMI**^b^, kg/m^2^; mean (SD)	27.0 (3.7)	26.2 (5.0)	27.4 (4.4)	26.6 (5.4)	0.021	0.075
**Education years**; % (n)					0.143	0.003
≤ 9	3.3 (21)	2.9 (21)	4.7 (142)	3.5 (127)		
9–12	73 (467)	71.3 (523)	72.9 (2222)	65.9 (2405)		
> 12	23.8 (152)	25.9 (190)	22.5 (685)	30.6 (1117)		
**Smoking**; % (n)					< 0.001	0.018
Non-smoker	48.8 (312)	59.8 (439)	44.7 (1349)	57.2 (2064)		
Former smoker	34.4 (220)	24.7 (181)	30.4 (917)	23.6 (853)		
Current smoker	16.9 (108)	15.5 (114)	24.9 (750)	19.2 (693)		
**Accelerometer data**						
Moderate to vigorous PA^c^, min/day; median (IQR^d^)	73 (53—97)	57 (40—76)	73 (52—97)	56 (41—76)	0.881	0.845
Wear time, min/day; median (IQR)	990 (948—1026)	972 (936—1008)	990 (948—1032)	978 (942—1008)	0.101	0.079
**Prevalence of MC**^e^; % (n)						
Any MC	74.4 (476)	59.7 (438)	75.2 (528)	60.1 (478)	0.098	0.455
MC1	32.7 (209)	31.7 (233)	32.4 (230)	31.0 (248)	0.708	0.113
MC2	65.3 (418)	44.4 (326)	65.7 (466)	44.3 (355)	0.479	0.858

^a^Standard deviations, ^b^Body mass index, ^c^Physical activity, ^d^Interquartile range, ^e^Modic changes

**Table 2:** Association between moderate to vigorous physical activity (MVPA) and Modic changes (MC) among men (*n* = 640) according to multivariable logistic regression models. Odds ratios (OR) correspond to every 60 min/day, i.e. 1 h per day, of MVPA

	Any MC			MC1			MC2		
	OR	95% CI^b^	*P*	OR	95% CI	*P*	OR	95% CI	*P*
Unadjusted	1.31	0.97, 1.78	0.080	1.06	0.81, 1.38	0.679	1.40	1.06, 1.85	0.018
Adjusted^a^	1.41	1.01, 1.95	0.042	1.06	0.80, 1.39	0.686	1.54	1.14, 2.08	0.005

^a^ Adjusted for BMI (body mass index, kg/m2), smoking, education years and accelerometer wear time

^b^ Confidence interval

**Table 3:** Association between moderate to vigorous physical activity (MVPA) and Modic changes (MC) among women (*n* = 734) according to multivariable logistic regression models. Odds ratios (OR) correspond to every 60 min/day, i.e. 1 h per day, of MVPA

	Any MC			MC1			MC2		
	OR	95% CI^b^	*P*	OR	95% CI	*P*	OR	95% CI	*P*
Unadjusted	1.07	0.80, 1.43	0.656	1.42	1.06, 1.92	0.020	0.88	0.66, 1.17	0.375
Adjusted^a^	1.23	0.89, 1.68	0.208	1.35	0.98, 1.85	0.068	1.04	0.76, 1.42	0.798

^a^ Adjusted for BMI (body mass index, kg/m2), smoking, education years and accelerometer wear time

^b^ Confidence interval

## Discussion

According to our present results, increased amount of MVPA was associated with increased odds of any MC (adjusted OR corresponding to every 60 min/day of MVPA 1.41; 95% confidence interval (CI) 1.01 to 1.95) and especially MC2 (OR 1.54; 95% CI 1.14 to 2.08) among men, whereas among women, MVPA was not independently associated with MC. In other words, among men, the odds of any MC increase by 41% and of MC2 by 54% if MVPA increased by 1 h/day. These findings strengthen the understanding of an association between PA and MC, especially MC2.

It is widely accepted that the same pathologic processes may lead to different types of MC, also suggesting that the more acute phase, i.e. Type 1, is interconvertible with Type 2, and that Type 3 is the late sclerotic phase [7–9]. Evidence from mixed types supports the interconvertibility of all types of MC [9, 14]. Various etiological factors leading to MC are debated. It has been suggested that MC1 results from bacterial infection and occult discitis, or an autoimmune reaction [1, 9, 29, 30], whereas MC2 is more strongly related to hyperloading and degenerative changes of the motion segment [9, 14, 31, 32]. Our current results are in line with these hypotheses, as we found no significant association between MVPA and MC1 among either sex in the adjusted models, but among men, increased amount of MVPA was positively associated with MC2, strengthening the hypothesis regarding a connection between hyperloading and MC2 [9, 14, 31, 32].

Biomechanical theory proposes that changed mechanical conditions in and around the intervertebral disc lead to microfractures of the vertebral endplate, which in turn, can lead to inflammatory processes seen as edema [1, 9, 20]. If mechanical stress and increased loading of the motion segment are considered potential contributors to the development of MC [1, 9, 20], intense PA could be hypothesized as a potential risk factor [33]. The findings concerning an association between PA and MC have been inconsistent [11, 14–16]. Kuisma et al. [14] studied the determinants of MC and found a significant association between questionnaire-based leisure-time PA and MC for all MC but not for MC1 or MC2 separately. Nevertheless, the authors speculated that mechanical loading due to high PA may damage endplates, thereby activating catabolic processes and resulting in MC.

In a Danish study, hard occupational PA, evaluated through questionnaires, was associated with MC among 40-year-olds, and the association was stronger among overweight participants and heavy smokers [15]. A more recent study [18] found a four times greater incidence of MC among study participants who did strenuous physical work than among participants who did light physical work. The authors speculated that the compression stresses and forces directed toward endplates during long-term strenuous physical work exceed the load-bearing capacity of endplates and result in damage that can gradually lead to the development of MC [18]. Our results are in line with the above-mentioned findings, as an increased amount of at least moderate intensity PA was associated with an increased likelihood of any MC and particularly MC2, although among women MVPA was not independently associated with MC.

Controversially, opposite findings have also been made [11, 16, 17, 34]. Jensen et al. [11] investigated the predictors of MC, i.e. high work and/or leisure-time PA, and concluded that PA did not predict MC. Arana et al. [16] similarly found no association between occupational or leisure-time PA and MC, but did find an association between DD and MC. More recent studies by Mok et al. [34] and Wu et al. [17] also investigated the relationship between MC and occupational and leisure-time PA, making the same conclusion of no association between PA and MC.

We found a significant association between increased amount of MVPA and MC among men but not among women. We do not know the reason for the sex difference, but speculate that men may be exposed to more hyperloading of the spine. The reason for the observed differences between the sexes has also remained indistinct in previous research. Leboeuf-Yde et al. [15] found no significant differences when analyzing the associations separately for men and women. Jensen et al. [11] found that gender did not predict MC, whereas Arana et al. [16] stated that male sex was associated with MC, and Han et al. [18] and Wu et al. [17] discovered an association between female sex and MC.

The strength of the present study is its large sample that included over 1300 individuals of the Northern Finnish population, thus increasing the relevance of the results when considering the applicability at the population level [35]. The representativeness analyses showed that the selected study participants were a representative sample of the total cohort population. Only small statistically significant but clinically non-relevant differences were found in BMI among men, smoking among both sexes and education years among women (Table 1). Thus, the groups were similar, and representativeness was good, as also demonstrated in our previous study [35].

The measurement of PA using an accelerometer can also be regarded as a strength of the study. In most previous studies investigating the association between PA and MC, PA assessment has relied on subjective self-reporting [11, 14–18, 34]. Currently, device-based measurement of PA is frequently preferred over subjective self-reporting of PA through, for example, questionnaires, as self-reporting of PA is prone to over- or underestimation of actual PA as well as recall and response bias. Meanwhile, accelerometers are considered proportionately sensitive and reliable, thus feasible for measuring PA [36–38].

Nevertheless, device-based monitoring of PA has its own limitations. Accelerometers cannot detect all varieties of motion, such as static activities (e.g. resistance training or cycling), and this may potentially lead to a biased estimation of total energy expenditure. Thus, the use of accelerometers can be also seen as a limitation [39–41]. However, the current trend is device-based measurement of PA and it is considered a more accurate measurement method than self-reporting of PA [39, 42]. We selected a wrist-worn over a waist-worn monitor as its wearing compliance has been found to be higher, and it has also been chosen by the National Health and Nutrition Examination Survey (NHANES) [43]. In the present study, the accelerometer was validated using the doubly labeled water technique. Good correlation for measuring energy expenditure during PA was detected (*R* = 0.86).

The cross-sectional study design can be considered the main limitation of this study as it prevents making conclusions about the causal relationship. The intensity of PA used in the assessments can also be seen as a limitation, as the association between MC and MVPA cannot be generalized to all PA. On the other hand, the possible beneficial role of light PA in bone health cannot be disregarded. There is broad discussion on the health effects of light PA of different intensities [44] as well as vibration exercises in terms of the prevention of falls and fractures [45]. Variety in PA can also be regarded as a limitation, as it makes it difficult to estimate the association of a specific activity with MC. Thus, we cannot contradict that lighter PA or specific activity could have even beneficial effects on lumbar MC. It can also be speculated whether a two-week period is truly representative of a person’s average activity level. However, earlier studies have indicated that monitoring of three to 7 days provides reliable estimates of PA behavior [42, 46–49]. Furthermore, an individual’s level of PA is assumed to be quite a stabile trait, meaning that the individuals who were physically active at the time of the study will presumably stay active during other periods of life [50]. Thus, it can be assumed that measuring PA over 2 weeks provides a reliable insight into the participants’ usual PA behavior.

## Conclusions

Among men, increased amount of PA reaching at least moderate intensity was associated with increased odds of any MC and particularly MC2; the odds of any MC increased by 41% and for MC2 by 54% if their MVPA increased by 1 h/day. Among women, MVPA was not associated with MC. It seems that PA may have clinical relevance among men in the form of increased likelihood of MC. Nevertheless, as the association between MC and LBP is still unclear, the clinical significance of the present results also remains debatable and further studies should be carried out to determine whether there is a meaningful and significant connection between PA and MC.

## Data Availability

The datasets used and analyzed during the current study are not publicly available due to local privacy regulations but are available from the corresponding author on reasonable request.

## Abbreviations

MC: Modic changes
LBP: Low back pain
PA: Physical activity
MVPA: Moderate-to-vigorous physical activity
MET: Metabolic equivalent
NFBC1966: Northern Finland Birth Cohort 1966
MRI: Magnetic resonance imaging
MC1: Type 1 Modic changes
MC2: Type 2 Modic changes
MC3: Type 3 Modic changes
BMI: Body mass index
OR: Odds ratio
95% Cl: 95% confidence interval
DD: Disc degeneration
EE: Energy expenditure
frFSE: Fast-recovery fast spin-echo
TR/effTE: Repetition time/effective echo time
FOV: Field-of-view
SD: Standard deviation
IQR: Interquartile range
